# Antioxidant and Hepatoprotective Effects of Procyanidins from Wild Grape (*Vitis amurensis*) Seeds in Ethanol-Induced Cells and Rats

**DOI:** 10.3390/ijms17050758

**Published:** 2016-05-18

**Authors:** Min Ji Bak, Van-Long Truong, Se-Yeon Ko, Xuan Ngan Giang Nguyen, Pajaree Ingkasupart, Mira Jun, Jin Young Shin, Woo-Sik Jeong

**Affiliations:** 1 Department of Smart Food and Drug, College of Biomedical Science & Engineering, Inje University, Gimhae 50834, Korea; bakmj0130@gmail.com (M.J.B.); truonglongpro@gmail.com (V.-L.T.); kose91@daum.net (S.-Y.K.); ngangiang.25590@gmail.com (X.N.G.N.); pajaree.ing@gmail.com (P.I.); 2 Department of Food Science and Nutrition, Dong-A University, Busan 49315, Korea; mjun@dau.ac.kr; 3 Chemistry and Environmental Science, City University of New York—Medgar Evers College, New York, NY 11225, USA; jinyoung.shin@njmeadowlands.gov

**Keywords:** procyanidins, alcohol, oxidative stress, hepatoprotection, CYP2E1, MAPKs

## Abstract

In the present study, we characterized the antioxidant and hepatoprotective mechanisms underlying of wild grape seed procyanidins (WGP) against oxidative stress damage in ethanol-treated HepG2 cell and Sprague-Dawley (SD)-rat models. In HepG2 cells, WGP not only diminished the ethanol (EtOH, 100 mM)-induced reactive oxygen species (ROS) formation and cytochrome P450 2E1 (CYP2E1) expression, but also renovated both the activity and expression of antioxidant enzymes including catalase, superoxide dismutase, and glutathione peroxidase. Additionally, to investigate the hepatoprotective effect of WGP, rats were orally administered 10 or 50 mg/kg WGP once daily for seven days prior to the single oral administration of EtOH (6 g/kg). The results show that WGP administration decreased the EtOH-induced augment of the levels of serum aspartate transaminase and alanine transaminase as well as serum alcohol and acetaldehyde. WGP treatment upregulated the activities and protein levels of hepatic alcohol dehydrogenase, aldehyde dehydrogenase, and antioxidant enzymes but downregulated the protein expression level of liver CYP2E1 in EtOH-treated rats. Moreover, the decreased phosphorylation levels of mitogen activated protein kinases (MAPKs) by ethanol were induced in both HepG2 cell and rat models. Overall, pretreatment of WGP displayed the protective activity against EtOH-mediated toxicity through the regulation of antioxidant enzymes and alcohol metabolism systems via MAPKs pathways.

## 1. Introduction

Different types of alcoholic beverages have played a vital part of the human diet since antiquity. However, long-term alcohol consumption is one of the major causes leading to serious liver diseases, such as liver cancer, fatty liver, and cirrhosis [1]. Mechanisms of ethanol-induced hepatotoxicity are associated to the metabolism of ethanol. The metabolic pathways involved in the hepatic transformation of ethanol are oxidation into acetaldehyde by alcohol dehydrogenase (ADH), microsomal ethanol oxidation system (MEOS), based on cytochromes P450 and, in particular, cytochrome P450 2E1 (CYP2E1), and to a much lesser extent by catalase (CAT) [2,3,4]. Acetaldehyde, a highly toxic metabolite of ethanol, is further metabolized to a less active by product acetate, which then is broken down into water and carbon dioxide [5]. Generation of reactive oxygen species (ROS) during ethanol metabolism is recognized to be a key step in the pathogenesis of ethanol-mediated liver tissue injury [6]. CYP2E1 is known as an effective generator of ethanol-mediated ROS formation, resulting in apoptotic cell death [7]. Under normal conditions, CYP2E1 accounts for 10% of ethanol oxidation, but it is inducible by chronic ethanol ingestion [8]. Enhanced CYP2E1 activity not only causes increased ROS formation but also results in an increased activation of various environmental pro-carcinogens that require CYP2E1 to be activated [3,9]. MEOS plays a major role in the oxidation of ethanol, as well as the formation of ROS, appearing to be mechanism for hepatotoxic-induced ethanol [10,11]. In addition to ROS, acetaldehyde accumulation by ADH- and CYP2E1-mediated ethanol metabolism is believed to contribute to ethanol-induced injury through DNA adducts formation, decreased DNA repair, and increased glutathione depletion. Therefore, CYP2E1 has been suggested to be a key contributor for the hepatotoxicity caused by alcohol and the resulting oxidative stress [12]. Recently, several *in vitro* and *in vivo* models using over-expression or knockouts of the *Cyp2e1* gene have been developed to examine the mechanisms of alcoholic liver diseases [13,14,15,16].

Accumulating evidence has suggested that acute and chronic ethanol administration results in increasing ROS production, reducing cellular antioxidant levels, and enhancing oxidative stress in various tissues, especially liver [17,18]. Alcohol-induced ROS can be attenuated by decomposing superoxide anions to hydrogen peroxide and hydrogen peroxide to water via the involvement of antioxidant enzymes including superoxide dismutase (SOD), glutathione peroxidase (GPx) and CAT [19,20]. Activation of antioxidant defense system is, therefore, regarded as a promising strategy to protect the liver against alcohol-induced oxidative damage.

Mitogen-activated protein kinases (MAPKs) including c-Jun N-terminal kinase (JNK), extracellular-regulated kinase (ERK), and p38 MAPK are well-known as signal transduction molecules that take part in the regulation of cell growth and differentiation, as well as cellular responses to various cellular stimuli [21]. Modulation of MAPK’s signaling pathway by ethanol depends on the cell/animal types, ethanol concentration and duration of exposure [22,23,24]. A variety of phytochemicals has been studied to protect cells and tissues against oxidative stress by the regulation of antioxidant enzymes and CYP2E1 via the modulation of MAPK signaling pathways [25,26,27].

Wild grape (*Vitis amurensis*) is a species of *Vitaceae* family distributed mainly in China, Russia, and Korea. Our group previously reported the isolation and identification of procyanidins from wild grape seeds (WGP) and demonstrated their chemopreventive properties through the PI3K/Akt-p38 MAPK-mediated activation of transcription factor Nrf2 (Nuclear factor erythroid-2 related factor 2) and phase II detoxifying/antioxidant enzymes [28]. WGP also effectively downregulated the inflammatory targets including inducible nitric oxide synthase (iNOS) and cyclooxygenase 2 (COX-2) by regulating the p38 MAPK and nuclear factor κ B (NFκB) pathways [29]. On the other hand, grape (*Vitis vinifera*) skins have been reported to exhibit the antioxidant effect in ethanol-induced mice [30]. Although various types of procyanidins from other species have been found to display radical scavenging antioxidant effects [31,32], little is known about antioxidant and hepatoprotective properties as well as the underlying mechanisms of WGP in the alcohol-induced hepatotoxicity model. In this present study, we examined the hepatoprotective and antioxidant mechanisms of WGP in alcohol-induced HepG2 cells and rats.

## 2. Results

### 2.1. Effect of Ethanol and Wild Grape Seed Procyanidins (WGP) on Viability of HepG2 Cells

**Figure 1:**
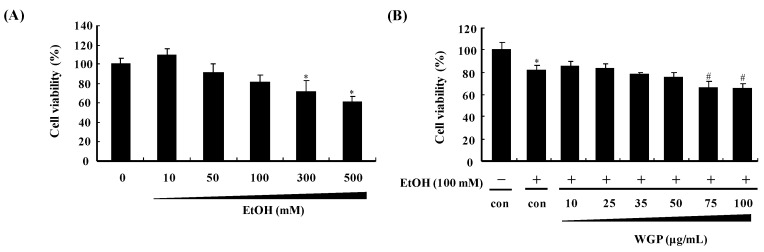
Cytotoxic effect of wild grape seed procyanidins (WGP) and ethanol on human hepatoma HepG2 cells. (**A**) Cells were treated with indicated concentration of ethanol (EtOH) for 24 h; and (**B**) cells were incubated with various concentrations of WGP in the presence of ethanol for 24 h. Cell viability was determined using MTT assay as described in materials and methods section. Cell viability was expressed as percentage of unstimulated-control cells. Values are means of three independent experiments ± SD (*n* = 3). * *p* < 0.05 indicates differences from the unstimulated-control group; ^#^ *p* < 0.05 indicates differences from the ethanol-treated group.

The cytotoxic effects of ethanol on HepG2 cell viability were evaluated using 3-(4,5-dimethythiazol-2-yl)-2,5-diphynyl tetrazolium bromide (MTT) assay. Cells were incubated with various concentrations of ethanol (10, 50, 100, 300, and 500 µM) for 24 h. The ethanol concentration at ≥100 mM produced a significant cell death in HepG2 cells; therefore, 100 mM ethanol was used for experiments to induce oxidative stress and CYP2E1 at 24 h (Figure 1A). In addition, the cytotoxic effects of WGP on viability of ethanol-treated HepG2 cells were measured and the result showed that WGP at concentrations up to 50 µg/mL was unlikely to change the cell survival compared to ethanol while higher doses of WGP resulted in decreases in cell viability (Figure 1B). Therefore, subsequent experiments were carried out with concentrations below 50 µg/mL of WGP.

### 2.2. Effect of WGP on the Cytochrome P450 2E1 (CYP2E1) Protein Level and Reactive Oxygen Species (ROS) Production in Ethanol-Treated HepG2 Cells

**Figure 2:**
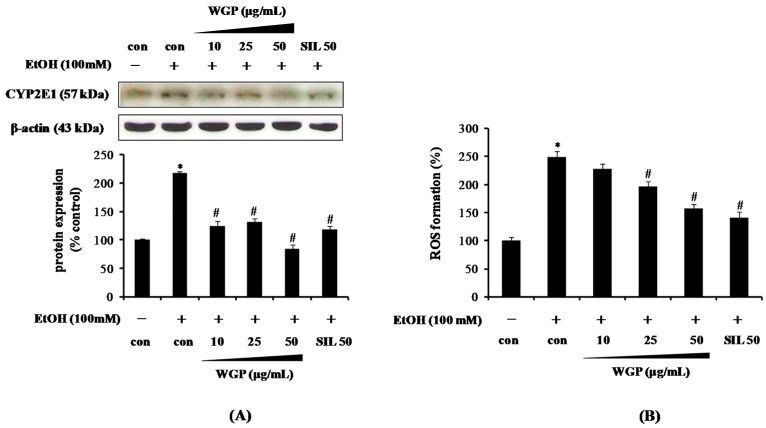
Effect of WGP on cytochrome P450 2E1 (CYP2E1) protein expression and reactive oxygen species (ROS) production in ethanol-treated HepG2 cells. (**A**) CYP2E1 protein expression; and (**B**) ROS production. Cells were pretreated with vehicle or WGP for 1 h before incubating with ethanol (100 mM) for 24 h. Values are the mean of three independent experiments ± SD (*n* = 3). * *p* < 0.05 indicates differences from the unstimulated-control group; ^#^ *p* < 0.05 indicates differences from the ethanol-treated group.

As shown in Figure 2A, the ethanol treatment resulted in the remarkable induction of CYP2E1 expression (>200%) compared with control. Pretreatment of the cells with WGP, however, diminished the ethanol-induced CYP2E1 protein level in a dose-dependent manner. In particular, WGP exhibited the more potent inhibition on CYP2E1 expression than the positive control silymarin (SIL) at the same concentration. To further examine whether the inhibitory effect of WGP on CYP2E1 expression is related to ethanol metabolism-mediated ROS formation, we measured intracellular ROS production using 2′,7′-dichlorofluorescin diacetate (DCF-DA) fluorescence at 24 h. The results indicated that ROS production increased about 250% by ethanol treatment compared to vehicle-treated control (Figure 2B). As expected, WGP pretreatment significantly and dose-dependently abolished the ethanol-induced intracellular ROS accumulation in the cells. A similar result was also observed from the positive control (SIL 50 µg/mL), which have been demonstrated as the most frequently used natural compound for the treatment of hepatic diseases due to its anti-oxidant anti-inflammatory activities. These results indicate that the inhibitory effect of WGP on the ethanol-mediated ROS production might be related to the ability of WGP to suppress of CYP2E1 expression.

### 2.3. Effect of WGP on the Activity and Protein Expression of Antioxidant Enzymes in Ethanol-Treated HepG2 Cells

**Figure 3:**
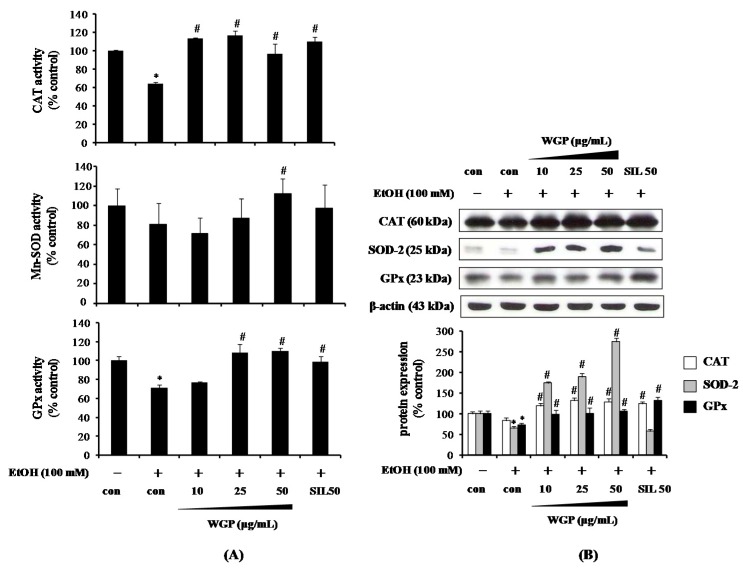
Effects of WGP on activity and protein expression of antioxidant enzymes in ethanol-treated HepG2 cells. (**A**) Activities of catalase (CAT), Mn-SOD, and glutathione peroxidase (GPx); and (**B**) protein levels of CAT, SOD-2, and GPx. Cells were pretreated with vehicle or WGP for 1 h before incubating with ethanol (100 mM) for 24 h. Data are the mean ± SD values of three individual experiments. * *p* < 0.05 indicates differences from the unstimulated-control group; ^#^ *p* < 0.05 indicates differences from the ethanol-treated group.

Antioxidant enzymes such as CAT, SOD, and GPx constitute the primary of enzymatic antioxidant defense system against oxidative stress through directly neutralizing ROS. Thus, we determined the effects of WGP on the activity and protein level of these antioxidant enzymes in ethanol-treated HepG2 cells. As illustrated in Figure 3A, 100 mM ethanol significantly reduced the activities of CAT, Mn-SOD, and GPx compared to vehicle-treated control, whereas pretreatment of cells with WGP or SIL restored the activity of antioxidant enzymes. In addition to the enzymes activity, the effect of WGP on the protein expression level of antioxidant enzymes were examined (Figure 3B). Protein levels of SOD-2 and GPx were effectively diminished by the treatment of ethanol 100 mM. Although not significant, protein expression of CAT slightly decreased when compared with untreated-control. For SOD-2, its induction by WGP was much greater than that by the positive control SIL. These results imply that WGP contributes to enhancement of antioxidant defense system against oxidative stress caused by ethanol metabolism via augmenting activity and expression level of antioxidant enzymes including CAT, SOD-2, and GPx.

### 2.4. Effect of WGP on Phosphorylations of Upstream Kinases in Ethanol-Treated HepG2 Cells

**Figure 4:**
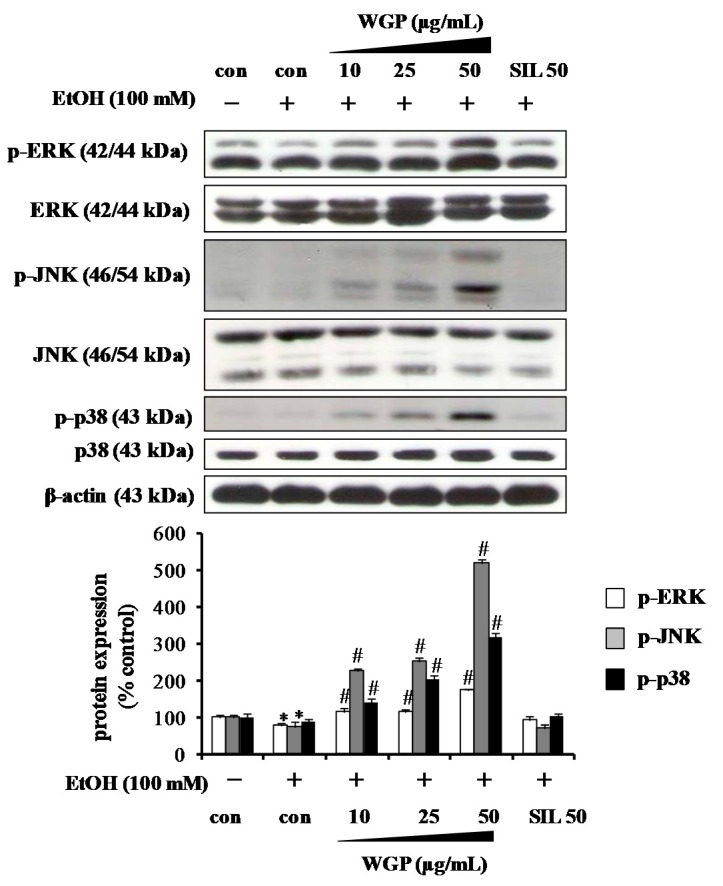
Effects of WGP on phosphorylation of mitogen activated protein kinases (MAPKs) in ethanol-treated HepG2 cells. Cells were pretreated with vehicle or WGP for 1 h before incubating with ethanol (100 mM) for 1 h. Values are the mean of three independent experiments ± SD (*n* = 3). * *p* < 0.05 indicates differences from the unstimulated-control group; ^#^ *p* < 0.05 indicates differences from the ethanol-treated group.

To elicit whether WGP can modulate the upstream signaling pathways, the phosphorylation levels of ERK, JNK and p38 MAPK were analyzed. Exposure of HepG2 cells to ethanol decreased phosphorylations of all MAPKs while WGP treatment elevated the phosphorylations of ERK, JNK, and p38 MAPK (Figure 4). However, WGP did not alter the protein level of MAPKs, suggesting that WGP modulated the activity of these kinases, but not the gene expression.

### 2.5. Protective Effect of WGP Against Hepatotoxicity in Ethanol-Treated Rats

**Figure 5:**
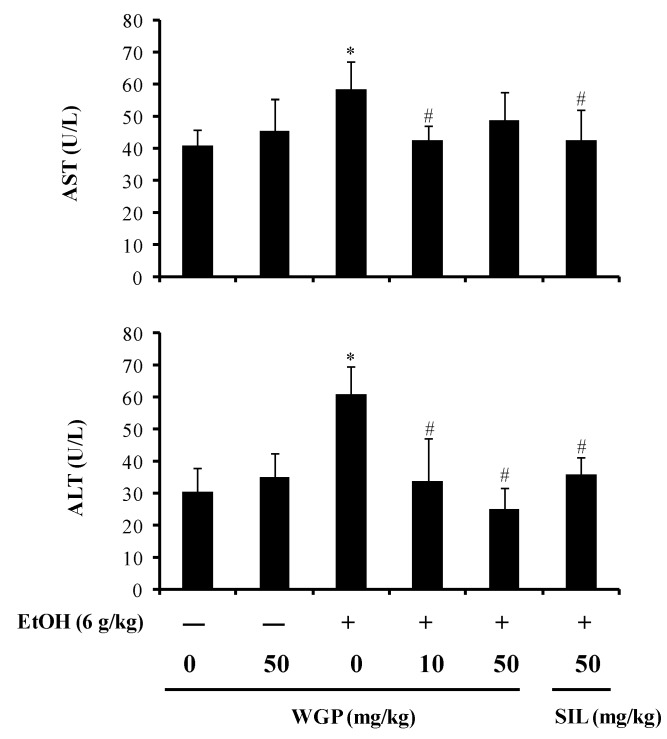
Effects of WGP administration on the serum levels of alanine aminotransferase (ALT) and aspartate aminotransferase (AST) in ethanol-treated Sprague-Dawley (SD)-rats. Each value represents the mean ± SD for five rats. * *p* < 0.05 indicates differences from the unstimulated-control group; ^#^ *p* < 0.05 indicates differences from the ethanol-treated group.

To further investigate the hepatoprotective effect of WGP against ethanol-induced toxicity *in vivo*, rats were received WGP at dose of 10 or 50 mg/kg for 7 days before a single administration of 6 mg/kg ethanol. As shown in Table 1, liver weights significantly increased in ethanol-treated group compared to vehicle-treated group and WGP alone-treated group, whereas the body weights were not significantly different among all groups. Along with the increased liver weight, the administration of ethanol also elevated the serum levels of alanine aminotransferase (ALT) and aspartate aminotransferase (AST), blood markers of liver damage (Figure 5). However, pre-oral administration of WGP significantly decreased the increase of ethanol-induced liver weight. Consequently, relative liver weight (liver/100 g body weight) was significantly diminished by the treatment of WGP. Additionally, enhanced levels of ALT and AST by ethanol were attenuated in the WGP group. SIL (50 mg/kg) was also found to reduce the liver weight and the plasma levels of ALT and AST. Moreover, treatment with 50 mg/kg WGP alone was unlikely to produce a significant alteration in the rat liver weight as well as the activity of transaminase enzymes, suggesting that no liver injury was caused by WGP.

### 2.6. Effect of WGP on the Ethanol Metabolism Pathway in Rats

**Figure 6:**
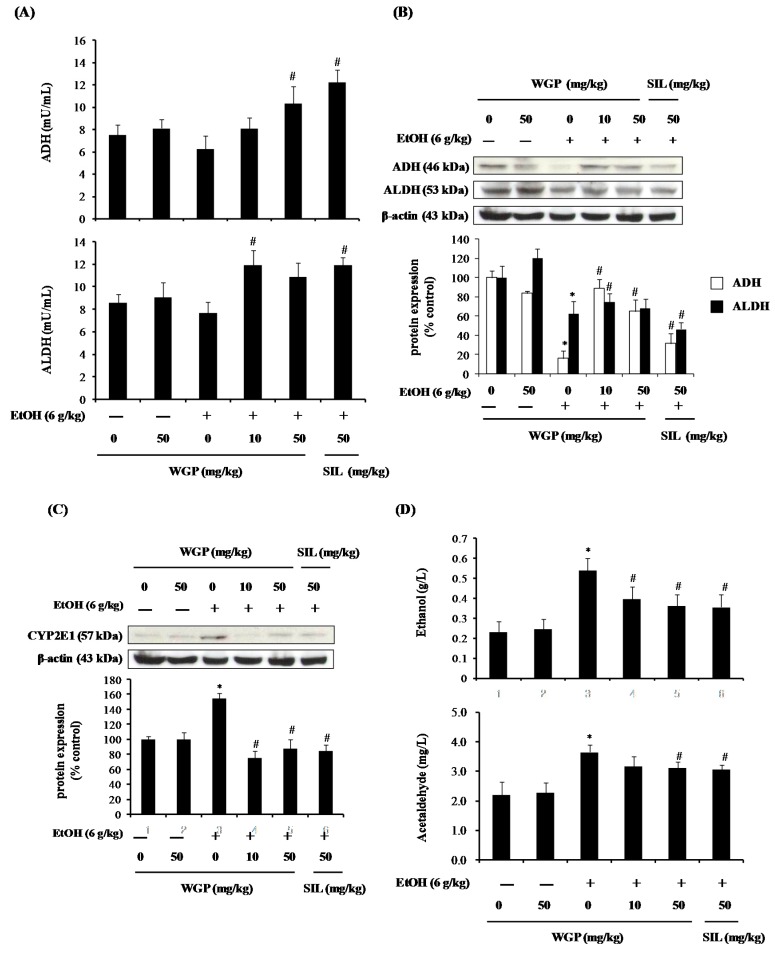
Effect of WGP administration on ethanol metabolism pathway in ethanol-treated SD-rats. (**A**) Serum levels of alcohol dehydrogenase (ADH) and aldehyde dehydrogenase (ALDH); (**B**) protein expressions of hepatic ADH and ALDH; (**C**) protein expression levels of hepatic CYP2E1; and (**D**) plasma levels of ethanol and acetaldehyde. Each value represents the mean ± SD for five rats. * *p* < 0.05 indicates differences from the unstimulated-control group; ^#^ *p* < 0.05 indicates differences from the ethanol-treated group.

Mechanisms of ethanol-induced hepatotoxicity are closely related to the metabolism of ethanol. Thus, the protein expression as well as activity of enzymes involving ethanol metabolism were evaluated. Ethanol-treated rats exhibited lower ADH and aldehyde dehydrogenase (ALDH) activities than the vehicle-treated rats (Figure 6A). Similarly, ethanol strongly abolished the protein expressions of ADH and ALDH in rat liver tissues when compared with the control (Figure 6B). In contrast, pretreatment of WGP or SIL restored the ethanol-decreased activities and protein levels of ADH and ALDH. CYP2E1 also involves in the ethanol oxidation leading to the production of acetaldehyde and to generation of ROS. Regulation of hepatic CYP2E1 expression by WGP was shown in the above mentioned cell line data as an evidence for its effect on ethanol-induced liver injury; thus, we examined the CYP2E1 expression in liver tissues in ethanol-treated rats. As displayed in Figure 6C, oral administration of ethanol to the rats produced a dramatic increase of the CYP2E1 expression in the liver. Pretreatment of rats with 10 or 50 mg/kg, however, suppressed the ethanol-elevated CYP2E1 protein level. Similar result was observed in SIL-treated group. Consequently, WGP-administered group exhibited a dose-dependent decrease of ethanol and acetaldehyde concentrations in plasma, whereas the serum ethanol and acetaldehyde still remain at high level in rats treated with ethanol alone (Figure 6D). Our data show that enhanced ADH and ALDH enzymes activity and suppressed CYP2E1 protein level by WGP importantly contribute to the eliminations of ethanol and acetaldehyde, as well as the reduction of oxidative stress.

### 2.7. Effect of WGP on the Activity and Protein Expression of Antioxidant Enzymes in Ethanol-Treated Rats

**Figure 7:**
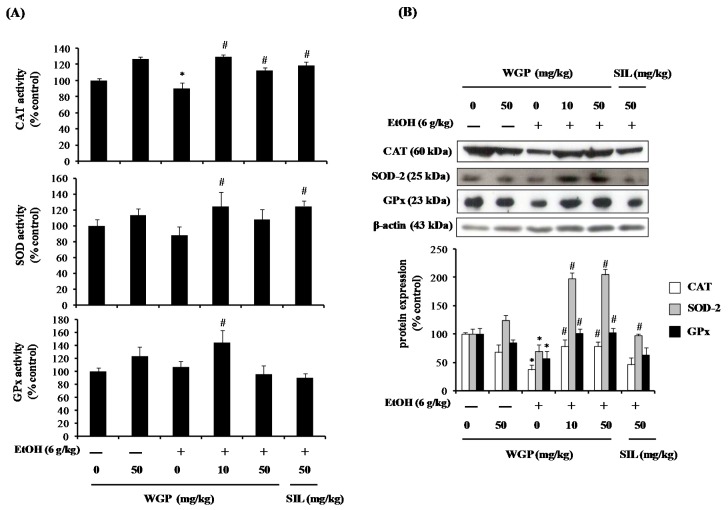
Effect of WGP on the activity and protein level of antioxidant enzymes in ethanol-treated SD-rats. (**A**) Activities of CAT, Mn-SOD, and GPx; and (**B**) protein expression levels of CAT, SOD-2, and GPx. Each value represents the mean ± SD for five rats. * *p* < 0.05 indicates differences from the unstimulated-control group; ^#^ *p* < 0.05 indicates differences from the ethanol-treated group.

The activity and expression level of antioxidant enzymes including CAT, Mn-SOD, and GPx by WGP were also determined in the ethanol-treated rats. As shown in Figure 7A, the activities of all three antioxidant enzymes were lowered in ethanol-treated group when compared to untreated-control group. However, pretreatment of rats with WGP at both 10 and 50 mg/kg reasonably restored the enzymes’ activity and similar results were obtained from the SIL-treated group. Along with enhanced enzyme activity, WGP reversed the inhibitory effect of ethanol on protein expression levels of CAT, SOD-2, and GPx in rat liver tissues (Figure 7B).

### 2.8. Effect of WGP on Phosphorylation of Upstream Kinases in Ethanol-Treated Rats

**Figure 8:**
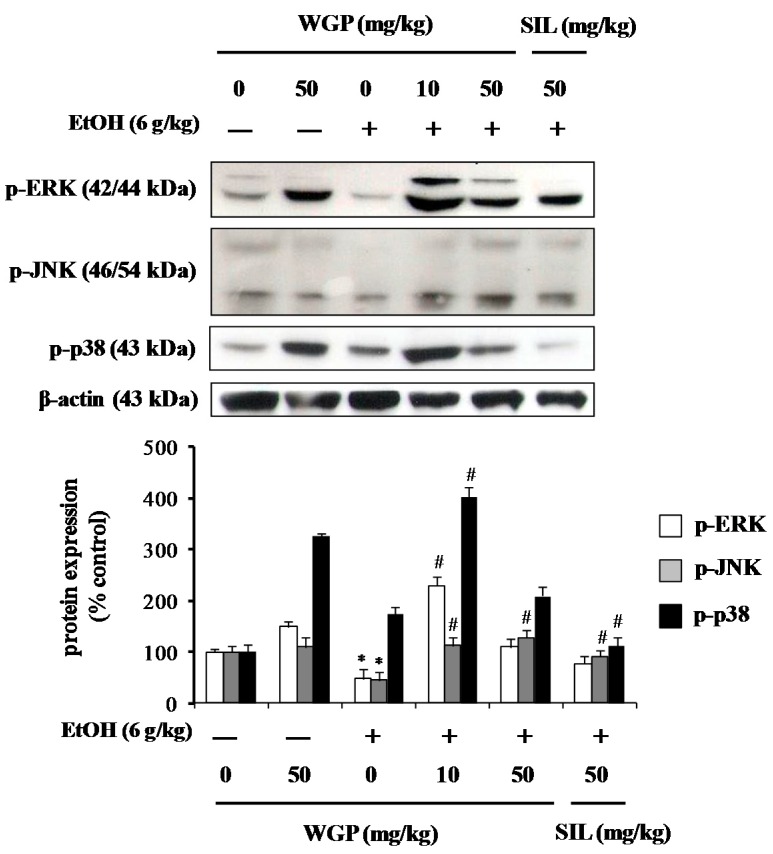
Effect of WGP on phosphorylation of MAP kinases in ethanol-treated SD-rats. Each value represents the mean ± S.D. for five rats. * *p* < 0.05 indicates differences from the unstimulated-control group; ^#^ *p* < 0.05 indicates differences from the ethanol-treated group.

To further examine the inductive mechanisms underlying the hepatic defense by WGP in ethanol-treated rats, the phosphorylation level of hepatic upstream MAP kinases were determined. As shown in Figure 8, the levels of phosphorylated ERK, JNK, and p38 MAPK were downregulated by ethanol treatment. Oral administration of rats with WGP resulted in the remarkably elevation of hepatic MAPKs phosphorylation. These results support those observed in the HepG2 cell model. This study proclaims, for the first time, the role of signal transduction pathways in antioxidant and hepatoprotective effects of WGP.

## 3. Discussion

Procyanidins, polymers of polyhydroxy flavan-3-ols, have long been explored as dietary supplements owing to their health promotion. Procyanidins are reported to enhance the activity of antioxidant enzymes and to protect against oxidative stress. Our previous studies isolated and demonstrated chemopreventive properties and anti-inflammatory effects of procyanidins from wild grape seeds and almond skins [28,29,33]. Grape seed procyanidins have also been indicated to protect the brain from ethanol-triggered oxidative DNA damage in mouse brain cells [34]. Although there are several reports on antioxidant and hepatoprotective effects of procyanidins, these studies only evaluated role of procyanidins on activity of antioxidant enzymes using different model systems and species of procyanidins. For the first time, our present study investigated the mechanisms underlying antioxidant and hepatoprotective effects of procyanidins from wild grape (*Vitis amurensis*) seeds in ethanol-induced cells, as well as in an animal model. Beneficial effects of procyanidins may be closely associated with their absorbability and bioavailability *in vivo*. Procyanidins up to pentamers have been indicated to be absorbed and present in blood circulation [35]. Moreover, polymeric procyanidins have been reported to be degraded by colonic microflora to oligomers with lower molecular weights, which are easily absorbed through intestinal wall [36]. Therefore, WGP with average polymerization degree of 4.65 could be, at least in part, absorbed and exhibit its hepatoprotective capacity in the current rat model.

Oxidative stress is a key factor of alcoholic liver diseases including alcoholic fatty liver, alcoholic hepatitis, fibrosis, cirrhosis, or cancer; therefore, the use of antioxidants and/or agents to induce antioxidant systems is a promising approach to reduce oxidative stress and alleviates oxidative damage as well as hepatotoxicity by ethanol. Our study demonstrated the role of WGP in the protection of cells/livers from ethanol-induced oxidative damage, possibly by the suppression of ROS formation, regulation of ADH, ALDH, and CYP2E1 enzymes, as well as the augmentation of the antioxidant defense system via MAPK upstream pathway in the HepG2 cells and SD-rats.

Ethanol consumption is known to induce CYP2E1, which not only leads to the increased generation of ROS, but also to the enhanced accumulation of carcinogen acetaldehyde, a major consequence of ethanol-induced hepatotoxicity [3]. In addition to ROS, acetaldehyde is also related to the formation of DNA adducts, the defect in DNA repair, and the increase of glutathione depletion. Therefore, CYP2E1 is considered to be a key enzyme responsible for ethanol-mediated oxidative stress, cell apoptosis and death, as well as liver injury. Our data confirmed the enhanced levels of ROS and CYP2E1 protein expression in ethanol-treated cells and rats. WGP provided a preventive potential against oxidative stress-related alcohol-induced liver damage through the inhibition of intracellular ROS accumulation and the down-regulation of CYP2E1 protein level. Similar with our results, ginkgolide B from *Ginkgo biloba* has been found to attenuate ethanol-induced toxicity through regulating ROS formation and CYP2E1 expression in PC12 cells [37]. Other recent studies also demonstrated that quercetin, catechin, caffeic acid, and phytic acid protected SK-HeP-1 cells from ethanol-induced oxidative stress by inhibiting ROS [38]. Abhilash *et al.* [39] reported that ascorbic acid and SIL prevented the ethanol-induced inflammation through the inhibitory effect on intercellular ROS production in hepatocytes of guinea pigs. Overexpression of CYP2E1 by chronic alcohol intake can cause overproduction of ROS, which decreases the level of ALDH leading to enhanced concentration of acetaldehyde, a highly toxic metabolite of ethanol [40]. Our results also showed that WGP could significantly increase both ALDH and ADH activities and expressions in the liver tissue, thereby rapidly pushing the conversion of ethanol into acetaldehyde and eventually forming acetate.

Antioxidant capacity can be reached by directly scavenging ROS/free radicals or indirectly activating cellular antioxidant defense systems including antioxidant enzymes [41]. The three major antioxidant enzymes including SOD, CAT, and GPx contribute to reduction of oxidative damage through the elimination of O_2_^−^ and H_2_O_2_ before their interaction to generate highly reactive hydroxyl radical [42]. Recently, their protective role has been clarified using *in vivo* and *in vitro* models [33,43]. Many studies demonstrated that ROS formation by chronic alcohol challenge resulted in decreased expressions of antioxidant enzymes including CAT, SOD, and GPx, contributing to enhancement of ethanol-caused toxicity [44,45]. Therefore, the activation of antioxidant enzymes plays an important role in protection of cells/tissues from ethanol-mediated CYP2E1-induced oxidative stress. WGP was likely to restore both the activity and protein level of hepatic antioxidant enzymes in ethanol-treated cells and rats. Previous reports have demonstrated that green tea polyphenols quercetin, EGCG, catechin, and betaine downregulate the CYP2E1 expression and upregulate GPx protein expression in HepG2 cells [46]. Liu *et al.* [47] reported that quercitrin pre-incubation resulted in the enhanced activity of antioxidant enzymes by preventing ethanol-mediated oxidative stress in hepatocytes. In addition, Noh *et al.* [25] showed that chestnut (*Castanea crenata*) inner shell extract exhibited the protective effects against ethanol-induced oxidative damage, possibly via suppressing lipid peroxidation and enhancing antioxidant enzymes in the C57BL/6 mice liver. In the present study, WGP exerts a regulatory effect on antioxidant enzymes and attenuates oxidative stress *in vitro* as well as *in vivo*.

MAPKs, such as ERK, JNK, and p38 MAPK, act as key players in regulation of signal transduction processes including proliferation, differentiation and apoptosis in response to various cellular stresses [48]. Involvement of MAPKs in the mechanisms of ethanol-induced hepatocellular injury is elsewhere. A recent study has revealed that Korean red ginseng and its ginsenosides reduce ethanol-induced oxidative injury through the inhibition of the MAPK pathway in mouse hepatocytes cell line TIB-73 [23]. Zeaxanthin and its derivatives are reported to prevent ethanol-induced hepatotoxicity by the inhibition of oxidative stress and inflammation through the upregulation of endogenous antioxidant enzyme and the downregulation of CYP2E1 protein along with the modulation of p38 MAPK and ERK pathways [49]. Jin *et al.* [45] have showed that PKC/JNK/SP1 pathway implicates in the regulation of CYP2E1 enzymes in ethanol-mediated oxidative stress, whereas Zeng *et al.* [50] has recently revealed that PI3K/Akt activation might be involved in acute ethanol-induced liver diseases in mice, but not with MAPK signaling. However, our results show that ethanol treatment results in the suppression of MAPKs in both *in vitro* and *in vivo* models. Additionally, the presence of WGP enhances the phosphorylation levels of ERK, JNK, and p38 MAPK in ethanol-treated cells and rats. The reason for such inconsistency is not clear at this moment and further studies are needed to elucidate exact molecular mechanisms involved in antioxidant and hepatoprotective effects by different agents, as well as the cell or tissues types.

## 4. Materials and Methods

### 4.1. Chemicals

Ethanol, nitroblue tetrazolium salt, xanthine, copper chloride, glutathione, xanthine oxidase from bovine milk (0.1–0.4 units/mg of protein), glutathione reductase from baker’s yeast (*Saccharomyces cerevisiae*, 100–300 units/mg of protein), and silymarin (SIL) were purchased from Sigma-Aldrich Co. (St. Louis, MO, USA). F-12 medium, fetal bovine serum (FBS) and penicillin/streptomycin were obtained from Hyclone (Logan, UT, USA). Antibodies against ADH, ALDH1/2, CYP2E1, SOD, GPx, β-actin, and horseradish peroxidase-conjugated anti-goat IgG were purchased from Santa Cruz Biotech (Santa Cruz, CA, USA). Anti-phospho-JNK, JNK, phospho-ERK, ERK, phospho-p38, p38, and horseradish peroxidase-conjugated anti-rabbit IgG were purchased from Cell Signaling technology Inc. (Beverly, MA, USA). CAT antibody was purchased from Abcam (Cambridge, MA, USA). All other chemicals in this study were analytical grade.

### 4.2. Preparation of WGP

Seeds of wild grape (*Vitis amurensis*) were generously supplied by Dooraemaeul Inc. (Hamyang, Korea). WGP were extracted from wild grape seeds and analyzed as described previously [28]. Briefly, 5 kg dried grape seeds powder was extracted in 70% aqueous acetone at room temperature. After 24 h, the aqueous acetone extract of wild grape seeds was partitioned with *n*-hexane to eliminate lipophilic components and fractionated with a Toyopearl HW-400F (Tosho, Tokyo, Japan) column using an aqueous solution of 50% methanol and 66% acetone and 100% acetone to obtain a procyanidins fraction. The procyanidins fraction was analyzed with various analytical techniques including the vanillin assay, butanol-HCl hydrolysis, and HPLC-MS analysis after depolymerization with phloroglucinol. The major procyanidins of WGP were determined as a mixture of prodelphinidins and procyanidins with the average polymerization degree of 6.22 and 4.65, respectively.

### 4.3. Cell Culture

HepG2 cells were purchased from American Type Culture Collections (ATCC, Rockville, MD, USA). Cells were cultured in F-12 medium containing 10% fetal bovine serum, 100 U/mL of penicillin/streptomycin, 1% essential amino acids, 1% glutamax and 0.1% insulin and maintained at 37 °C in a humidified CO_2_ incubator.

### 4.4. Animal Experimental Design

Male Sprague-Dawley (SD) rats, five weeks old (weighting 170–190 g), were obtained from Hyochang Science (Daegu, Korea) and housed in stainless cages under a condition of humidity of 50% ± 5%, a controlled temperature room (22 ± 2 °C), and a 12/12 h of light/dark. Rats were supplied with standard food and water *ad libitum*. This animal experiment was approved by the Animal Care and Used Committee of Inje University with protocol number 2015-18 (Gimhae, South Korea). Rats were randomly grouped (six groups, *n* = 5 per group) and treated as following manner: Group I (normal control group) daily received polyethylene glycol (PEG) for seven days, followed by saline; Group II received WGP at 50 mg/kg orally once daily for seven days, followed by saline; Group III (negative control group) daily administered PEG for seven days, followed by a single dose of ethanol (6 g/kg, 20% *w*/*v* per os (p.o.)); Group IV and V orally received WGP at 10 or 50 mg/kg daily for seven days, respectively, followed by a single dose of ethanol (6 g/kg, 20% *w*/*v* p.o.); Group VI was orally administered 50 mg/kg of SIL every day for seven days, followed by a single dose of ethanol (6 g/kg, 20% *w*/*v* p.o.). All the animals were humanely sacrificed after the last dose administration for 6 h. The serum was obtained by centrifugation at 3000× *g* for 15 min. The livers were harvested immediately, washed with ice-cold saline, and stored at −80 °C for further experiments.

### 4.5. Cell Viability Assay

Cell viability was determined using MTT assay, which is the reduction of yellow 3-(4,5-dimethythiazol-2-yl)-2,5-diphynyl tetrazolium bromide (MTT) by mitochondrial succinate dehydrogenase in viable cells. Briefly, HepG2 cells were cultured in a 24-well plate (1 × 10^5^ cells/well) for 24 h and then starved in serum-free media overnight. A series of various concentrations of samples (10, 50, 100, 300, and 500 µM for EtOH, and 10, 25, 35, 50, 75, and 100 µg/mL for WGP) was added to each well and cultured for 24 h, followed by incubation with 5 mg/mL MTT for 4 h in darkness. The supernatant was removed, and the formazan crystal in each well was dissolved in DMSO for 1 h at room temperature in darkness. The absorbance was read at 570 nm on a microplate reader with an ELISA reader from BioTek Instruments (Winooski, VT, USA). Cell viability was expressed as a percentage of the vehicle-treated control.

### 4.6. ROS Formation Assay

Intracellular ROS levels were measured using a DCF-DA assay as previously described [51]. Cells were seeded in 96-well dark plates (1 × 10^4^ cells/well). After starvation with serum-free medium, the cells were treated vehicle or WGP in the presence of absence of 100 mM ethanol for 24 h and then washed twice with ice-cold phosphate-buffered saline (PBS). The cells were incubated with 20 µM DCF-DA for 1 h at 37 °C in darkness. After washing with PBS twice, the detection was performed using a fluorescence multi-detection reader (BioTek Instruments, Winooski, VT, USA) with excitation at 485 nm and emission at 535 nm.

### 4.7. Assessment of Antioxidant Enzymes Activity in HepG2 Cells and Liver Tissues

Cells were plated in 6-well plates at a rate of 2 × 10^5^ cells per well and treated with 10, 25, and 50 µg/mL of WGP for 1 h and followed by treatment with ethanol for 24 h. The cells and liver tissues were then homogenized in appropriate buffer, as follows. After centrifugation, the supernatant was used to examine the enzyme activity of CAT and GPx, while the pellet was subjected to determine Mn-SOD activity. The protein concentration was analyzed by the BCA protein assay (Pierce Biotechnology, Rockford, IL, USA) according to the manufacturer’s protocols. Enzyme activity was normalized with a protein concentration and expressed as the fold factor of enzyme activity over the vehicle-treated control. Mn-SOD activity was analyzed by the modified method of Oyanagui [52]. The remaining pellet was lysed in 0.1%Triton and used to measure Mn-SOD activity. 4 mM KCN was added to the assay mixture to inhibit Cu/Zn-SOD. The mixtures containing supernatant, 75 mM Na-xanthine, and 10 mM hydroxylamine hydrochloride were pre-incubated at 37 °C for 10 min. Then 0.1 units of xanthine oxidase were added to mixtures and incubated at 37 °C for an additional 20 min, and the reaction was terminated by the addition of 1% sulfanilamide and 0.02% ethylenediamine dihydrochloride for 20 min. The absorbance of the final mixture was determined at 450 nm. CAT activity was evaluated by modified the method of Carrillo *et al.* [53]. The reaction mixtures containing 3% (*v*/*v*) H_2_O_2_ and 20 µL of cell lysates in 50 mM potassium phosphate buffer (pH 7.0) at a final volume of 1.0 mL were incubated for 5 min at 37 °C and the absorbance of each sample was read for 5 min at 240 nm. The change in absorbance is proportional to the breakdown of H_2_O_2_. GPx activity was analyzed by modified the method of Bogdanska *et al*. [54]. The reaction mixture consisted of 0.1 M phosphate buffer (pH 7.0), 1 mM EDTA, 5 mM glutathione, 1 mM NaN_3_, 1 unit of glutathione reductase, and 1 mM NADPH and cell supernatant. The reaction was initiated by the addition of 2.5 mM H_2_O_2_. The activity was calculated using the molar extinction coefficient for NADPH of 6.22 µmol/cm at 340 nm.

### 4.8. Assays of ADH and ALDH Activities

The ADH and ALDH concentrations were evaluated with detection kit (Biovision, Milpitas, CA, USA) according to the manufacturer’s instructions, and the optical densities were measured at 450 nm with an ELISA reader from BioTek Instruments (Winooski, VT, USA). The optical densities were then converted to concentrations of ADH and ALDH by using nicotinamide adenine dinucleotide (NADH) standard curves.

### 4.9. Measurements of Alcohol and Acetaldehyde Concentrations

The alcohol and acetaldehyde levels were measured using a detection kit (Biovision, Milpitas, CA, USA) according to the manufacturer’s instructions. The concentrations of alcohol and acetaldehyde were calculated by using alcohol and acetaldehyde standards. The optical densities were determined with an ELISA reader (BioTek Instruments, Winooski, VT, USA) at 570 nm.

### 4.10. AST and ALT Activities

Serum levels of AST and ALT levels were determined using commercially available kits (Young-Dong Co., Seoul, Korea).

### 4.11. Western Blot Analysis

After treatment, cells were washed twice with ice-cold PBS (pH 7.4) and harvested in 1× whole cell lysis buffer. In rats, liver tissues were homogenized in cell lysis buffer. The collected cells and homogenized liver tissues were centrifuged at 13,000 rpm to obtain supernatants. The protein concentrations were analyzed by the BCA protein assay (Pierce) reagent based on the manufacturer’s protocols. Equal amounts of proteins were loaded onto a 12% SDS-polyacrylamide for gel electrophoresis and then transferred onto a PVDF membrane (Bio-Rad, Hercules, CA, USA) for 1 h using a semi-dry transfer system (Bio-Rad). The membrane was blocked with 5% nonfat milk in PBST buffer (0.1% Tween 20 in PBS) for 1 h at room temperature and then incubated overnight with the appropriate primary antibodies. After hybridization with primary antibody, the membrane was washed five times with PBST, and then incubated with anti-goat or anti-rabbit IgG horseradish peroxidase-conjugated secondary antibodies for 1 h at room temperature and washed with PBST five times. Final detection was performed with Western Blotting Luminol Reagents (Santa Cruz Biotechnology, Santa Cruz, CA, USA).

### 4.12. Statistical Analysis

The data were expressed as mean ± standard deviation (SD) by Microsoft Excel (Microsoft Corp., Washington, DC, USA). Student’s *t*-test was used to examine the statistically significant difference between groups. *p*-value of <0.05 was considered statistically significant.

## 5. Conclusions

In conclusion, the present study indicated that WGP exerts the protective ability of cells/tissues against ethanol-induced oxidative damage by directly scavenging ROS, regulating ADH and ALDH enzymes, and inhibiting CYP2E1 expression, as well as inducing both activity and expression of cellular antioxidant enzymes through activating MAPK signaling pathways. Taken together, our results suggested that WGP may be a potential therapeutic agent for prevention of the ethanol-induced liver damage.

## Figures and Tables

**Table 1:** Effect of wild grape seed procyanidins (WGP) on body weight and liver weight/body weight in ethanol-treated Sprague-Dawley (SD)-rats. Each value represents the mean ± SD for five rats.

Groups	Initial Body Weight (g)	Final Body Weight (g)	Liver Weight (g)	Relative Liver Weight (Liver/100 g Body Weight)
Control	188.94 ± 5.61	311.34 ± 9.17	12.7 ± 0.55	4.08 ± 0.11
WGP (50 mg/kg)	171.80 ± 6.12	315.67 ± 9.95	13.01 ± 2.16	4.17 ± 0.90
Ethanol (EtOH) (6 g/kg)	179.18 ± 3.69	312.30 ± 13.69	16.10 ± 2.06 *	5.18 ± 0.86 *
WGP (10 mg/kg) + EtOH	180.84 ± 6.26	313.65 ± 12.65	13.20 ± 1.84 ^#^	4.20 ± 0.51 ^#^
WGP (50 mg/kg) + EtOH	178.28 ± 6.80	320.62 ± 14.22	13.20 ± 0.83 ^#^	4.12 ± 0.16 ^#^
Silymarin (SIL) (50 mg/kg) + EtOH	179.92 ± 5.42	323.75 ± 8.48	12.78 ± 1.71 ^#^	3.94 ± 0.46 ^#^

* *p* < 0.05 indicates differences from the unstimulated-control group; ^#^ *p* < 0.05 indicates differences from the ethanol-treated group.

## Abbreviations

ADH	alcohol dehydrogenase
ALDH	aldehyde dehydrogenase
ALT	alkaline phosphatase
AST	aspartate aminotransferase
CAT	catalase
CYP2E1	cytochrome P450 2E1
DCF-DA	2′,7′-dichlorodihydrofluorescein
ERK	extracellular regulated kinase
GPx	glutathione peroxidase
JNK	c-Jun N-terminal kinase
MAPK	mitogen-activated protein kinase
ROS	reactive oxygen species
SIL	silymarin
SOD	superoxide dismutase
WGP	wild grape seeds procyanidins
